# Supplementary material for: MetaRibo-Seq measures translation in microbiomes
Source: Nat Commun. 2020 Jun 29;11:3268. doi: 10.1038/s41467-020-17081-z (PMC7324362; doi:10.1038/s41467-020-17081-z)
Supplement: Supplementary file 10 — Supplementary Data 7 [file 41467_2020_17081_MOESM10_ESM.zip › File2/Confidence_High_Taxonomy/332687_out.krona.html]

Javascript must be enabled to view this page.

members
magnitude
magnitudeUnassigned
count
unassigned
taxon
rank

332687\_out

193

superkingdom
2
193

phylum
1239
190

1262992
1

SRS148253\_contig\_number\_9354
species


SRS014235\_contig\_number\_contig-100\_1253.257459
species
1
1263002

class
186801
188

order
186802
188


SRS013098\_contig\_number\_contig-100\_39.218902SRS013940\_contig\_number\_5567
species
1898207
2

186803
1
family

1
841
genus

1
360807
species

SRS015431\_contig\_number\_72518

541000
9
family

1
216851
genus

1
259315

SRS143148\_contig\_number\_13506
species

species

SRS024663\_contig\_number\_9512SRS077294\_contig\_number\_10733SRS077641\_contig\_number\_20884SRS143070\_contig\_number\_3477SRS147271\_contig\_number\_10586
2292270
5

2
2292180
species

SRS058145\_contig\_number\_contig-100\_3455.38058SRS077991\_contig\_number\_contig-100\_1355.1356

1
1898205
species

SRS893279\_contig\_number\_15839

family
216572
175

459786
175
genus


SRS015890\_contig\_number\_23348
species
1
1897011

species

SRS011084\_contig\_number\_11110SRS011134\_contig\_number\_40298SRS012849\_contig\_number\_25617SRS013382\_contig\_number\_148SRS013521\_contig\_number\_contig-100\_438.92902SRS013687\_contig\_number\_26143SRS013951\_contig\_number\_30400SRS014412\_contig\_number\_4452SRS014415\_contig\_number\_contig-100\_347.348SRS014459\_contig\_number\_39829SRS014855\_contig\_number\_8117SRS014923\_contig\_number\_44188SRS014948\_contig\_number\_3477SRS015065\_contig\_number\_37007SRS015190\_contig\_number\_6407SRS015579\_contig\_number\_24673SRS015663\_contig\_number\_14454SRS015694\_contig\_number\_22105SRS015782\_contig\_number\_45251SRS015960\_contig\_number\_contig-100\_2262.122376SRS016018\_contig\_number\_6315SRS016056\_contig\_number\_7022SRS016095\_contig\_number\_26940SRS016203\_contig\_number\_4499SRS016335\_contig\_number\_41096SRS016753\_contig\_number\_6195SRS017103\_contig\_number\_31735SRS017191\_contig\_number\_19521SRS017307\_contig\_number\_29550SRS017821\_contig\_number\_29766SRS017916\_contig\_number\_10117SRS018351\_contig\_number\_3801SRS018541\_contig\_number\_9573SRS018575\_contig\_number\_4947SRS018623\_contig\_number\_12075SRS018656\_contig\_number\_11648SRS018836\_contig\_number\_17221SRS018888\_contig\_number\_2786SRS019030\_contig\_number\_7533SRS019397\_contig\_number\_9827SRS019445\_contig\_number\_16633SRS019496\_contig\_number\_10142SRS019685\_contig\_number\_34144SRS019693\_contig\_number\_8413SRS019808\_contig\_number\_17761SRS019968\_contig\_number\_14146SRS020233\_contig\_number\_40529SRS020271\_contig\_number\_contig-100\_2004.2005SRS021484\_contig\_number\_30614SRS022137\_contig\_number\_16939SRS022609\_contig\_number\_40465SRS024075\_contig\_number\_11672SRS024331\_contig\_number\_14147SRS024435\_contig\_number\_40115SRS024625\_contig\_number\_17865SRS042628\_contig\_number\_25296SRS043411\_contig\_number\_17394SRS043701\_contig\_number\_16286SRS043768\_contig\_number\_14832SRS043841\_contig\_number\_4261SRS044535\_contig\_number\_19872SRS045004\_contig\_number\_contig-100\_10780.127017SRS045713\_contig\_number\_11047SRS045739\_contig\_number\_11143SRS045826\_contig\_number\_1629SRS046502\_contig\_number\_11233SRS047014\_contig\_number\_23238SRS047433\_contig\_number\_19103SRS048164\_contig\_number\_30290SRS049446\_contig\_number\_13494SRS049896\_contig\_number\_17685SRS049995\_contig\_number\_14163SRS050422\_contig\_number\_27812SRS050925\_contig\_number\_34626SRS050998\_contig\_number\_14949SRS051031\_contig\_number\_34500SRS053398\_contig\_number\_14893SRS053573\_contig\_number\_contig-100\_375.37947SRS054059\_contig\_number\_4599SRS054352\_contig\_number\_13124SRS054905\_contig\_number\_21001SRS055982\_contig\_number\_6248SRS056259\_contig\_number\_contig-100\_13790.113516SRS056273\_contig\_number\_22238SRS056519\_contig\_number\_11432SRS057480\_contig\_number\_8483SRS058070\_contig\_number\_14344SRS062654\_contig\_number\_21344SRS062701\_contig\_number\_5065SRS063040\_contig\_number\_27638SRS063370\_contig\_number\_6911SRS063518\_contig\_number\_30951SRS063985\_contig\_number\_contig-100\_3255.144561SRS064276\_contig\_number\_19976SRS064757\_contig\_number\_21276SRS065397\_contig\_number\_5869SRS074586\_contig\_number\_contig-100\_1591.42364SRS074670\_contig\_number\_8349SRS074964\_contig\_number\_6085SRS075078\_contig\_number\_773SRS075341\_contig\_number\_24532SRS075773\_contig\_number\_34979SRS075821\_contig\_number\_11870SRS075878\_contig\_number\_18149SRS075984\_contig\_number\_27620SRS076756\_contig\_number\_16838SRS076804\_contig\_number\_6781SRS076929\_contig\_number\_14531SRS077127\_contig\_number\_11416SRS077231\_contig\_number\_24676SRS077552\_contig\_number\_27705SRS078176\_contig\_number\_contig-100\_1208.135222SRS078242\_contig\_number\_28952SRS097920\_contig\_number\_6103SRS098073\_contig\_number\_16676SRS098571\_contig\_number\_40389SRS098644\_contig\_number\_24873SRS098717\_contig\_number\_14089SRS098881\_contig\_number\_9207SRS100907\_contig\_number\_1001SRS101376\_contig\_number\_3909SRS101433\_contig\_number\_14677SRS103987\_contig\_number\_28906SRS104036\_contig\_number\_contig-100\_2615.42400SRS1041037\_contig\_number\_11011SRS1041092\_contig\_number\_10856SRS1041116\_contig\_number\_contig-100\_759.47146SRS1041134\_contig\_number\_6303SRS1041141\_contig\_number\_11001SRS1041142\_contig\_number\_6779SRS104311\_contig\_number\_22527SRS104400\_contig\_number\_46422SRS104693\_contig\_number\_3815SRS104912\_contig\_number\_18888SRS104975\_contig\_number\_21101SRS1055022\_contig\_number\_18670SRS1055067\_contig\_number\_5402SRS1055083\_contig\_number\_13694SRS1055099\_contig\_number\_11177SRS140513\_contig\_number\_contig-100\_478.478SRS142503\_contig\_number\_55807SRS142542\_contig\_number\_25026SRS142599\_contig\_number\_19092SRS142890\_contig\_number\_21475SRS142923\_contig\_number\_16246SRS143085\_contig\_number\_5981SRS143181\_contig\_number\_14416SRS143342\_contig\_number\_17189SRS143417\_contig\_number\_20065SRS143523\_contig\_number\_24077SRS143598\_contig\_number\_6078SRS143895\_contig\_number\_2514SRS143991\_contig\_number\_19583SRS144362\_contig\_number\_27679SRS144506\_contig\_number\_23988SRS144603\_contig\_number\_12504SRS145497\_contig\_number\_13990SRS147346\_contig\_number\_63059SRS147614\_contig\_number\_2171SRS148159\_contig\_number\_48970SRS148319\_contig\_number\_17289SRS148721\_contig\_number\_8830SRS148817\_contig\_number\_12668SRS149325\_contig\_number\_11549SRS150029\_contig\_number\_9760SRS893170\_contig\_number\_755SRS893231\_contig\_number\_5883SRS893292\_contig\_number\_9524SRS893295\_contig\_number\_10910SRS893300\_contig\_number\_contig-100\_69.75407SRS893373\_contig\_number\_21582SRS893378\_contig\_number\_12265SRS893383\_contig\_number\_contig-100\_2361.194708SRS971275\_contig\_number\_7695
174
1945593

172733
1

SRS058070\_contig\_number\_16980
species

phylum
1224
1

class
28216
1

80840
1
order

995019
1
family

genus
40544
1


SRS104327\_contig\_number\_2325
species
40545
1

phylum
976
2

200643
2
class

order
2
171549

family
2
171550

239759
2
genus

species

SRS104084\_contig\_number\_9613
1
214856

species

SRS011302\_contig\_number\_30395
1
28117
